# Science policies: How should science funding be allocated? An evolutionary biologists’ perspective

**DOI:** 10.1111/jeb.13497

**Published:** 2019-07-05

**Authors:** Stephanie Meirmans, Roger K. Butlin, Anne Charmantier, Jan Engelstädter, Astrid T. Groot, Kayla C. King, Hanna Kokko, Jane M. Reid, Maurine Neiman

**Affiliations:** ^1^ Amsterdam UMC University of Amsterdam Amsterdam The Netherlands; ^2^ Department of Animal and Plant Sciences The University of Sheffield Sheffield UK; ^3^ Department of Marine Sciences University of Gothenburg Strömstad Sweden; ^4^ CEFE UMR 5175 CNRS Université Paul‐Valery Montpellier Université de Montpellier Montpellier Cedex 05 France; ^5^ School of Biological Sciences The University of Queensland Brisbane Queensland Australia; ^6^ Institute for Biodiversity and Ecosystem Dynamics (IBED) University of Amsterdam Amsterdam The Netherlands; ^7^ Department of Zoology Christ Church College University of Oxford Oxford UK; ^8^ Department of Evolutionary Biology and Environmental Studies University of Zurich Zurich Switzerland; ^9^ School of Biological Sciences University of Aberdeen Aberdeen UK; ^10^ Department of Biology University of Iowa Iowa City IA USA; ^11^ Department of Gender, Women's, and Sexuality Studies University of Iowa Iowa City IA USA

**Keywords:** funding, funding rate, grant proposal, science policy, scientific quality

## Abstract

In an ideal world, funding agencies could identify the best scientists and projects and provide them with the resources to undertake these projects. Most scientists would agree that in practice, how funding for scientific research is allocated is far from ideal and likely compromises research quality. We, nine evolutionary biologists from different countries and career stages, provide a comparative summary of our impressions on funding strategies for evolutionary biology across eleven different funding agencies. We also assess whether and how funding effectiveness might be improved. We focused this assessment on 14 elements within four broad categories: (a) topical shaping of science, (b) distribution of funds, (c) application and review procedures, and (d) incentives for mobility and diversity. These comparisons revealed striking among‐country variation in those elements, including wide variation in funding rates, the effort and burden required for grant applications, and the extent of emphasis on societal relevance and individual mobility. We use these observations to provide constructive suggestions for the future and urge the need to further gather informed considerations from scientists on the effects of funding policies on science across countries and research fields.

## 1 FUNDING CRISIS: WHAT ARE THE CRITICAL CHALLENGES AND HOW CAN THESE CHALLENGES BE ADDRESSED?

Scientific funding agencies would ideally be able to select excellent scientists and research projects and provide these scientists with sufficient resources to undertake the best possible work. Indeed, these goals should constitute the ultimate aspiration of any funding programme. Many countries recognize that investment in scientific research is central to economic and societal advances, manifested as substantial government investment of GDP in science (OECD, 2018; Stephan, 2012). Nevertheless, because financial limitations often impose severe constraints on the ability of funding agencies to support excellent scientists and their ideas, deeper understanding of how to allocate funding most effectively is of critical importance.

It is obvious that finite funding supply can prevent the execution of at least some excellent research. There are additional negative consequences of the imbalance between the intellectual capacity of the scientific community and available resources (see also, e.g., Stephan, 2012; Alberts, Kirschner, Tilghman, & Varmus, 2014; Franssen, Scholten, Hessels, & de Rijcke, 2018; Whitley, Gläser, & Laudel, 2018). For example, the high workload connected to the need to submit many grant proposals to achieve funding success can in turn generate high stress levels, increasing despondency, frustration and lack of motivation. These issues can be exacerbated for junior scientists, whose careers often depend on the acquisition of external funding prior to gaining a permanent faculty position (Powell, 2016). Proposal reviewing and administrative burdens also tend to be heavier when funding is limited, with researchers often forced to submit more applications as funding rates decrease. A further decrease in funding rates will result from this negative cycle. Intense competition for funding can also generate downstream negative consequences ranging from the abandonment of promising but risky ideas in favour of more “fundable” projects (Fochler, Felt, & Müller, 2016; Laudel, 2006; Powell, 2016; Stephan, 2012) to the incentivization of questionable research practices and even fraudulent behaviour (Moore, Neylon, Eve, O'Donnell, & Pattinson, 2017; Tijdink et al., 2016).

In our opinion, low funding rates underlie a transition from “eustress,” the positive stress state associated with healthy fair competition for limited resources, to “distress,” the negative destructive stress state, in many countries. Eustress in this context can arise because some degree of fair competition can help generate motivation, and because regular and clear statements and peer evaluation of research goals and project plans help to maintain and increase scientific quality. Distress can be generated when resource restriction is so severe that the funding system becomes dysfunctional and impedes rather than promotes scientific quality and progress. Our evaluation suggests that this distress state now characterizes the scientific community in multiple countries, culminating in waste of precious available resources and failure to maximize the potential for rapid scientific progress (see also, e.g., Alberts et al., 2014).

In our view, the consequences of severe funding limitation extend beyond the applicants (see also Stephan, 2012). First, administration of the applications uses a substantial fraction of available resources (see also Vaesen & Katzav, 2017). Second, peer reviewers and committee members might feel that they can no longer make a useful contribution, leading to a “distress” state involving substantial waste of time, effort and financial resources linked to a lengthy process of application, review and re‐review before worthy projects are funded. Although grant writing can be helpful when causing "eustress", as it makes researchers think about the next research question and how to approach it, in a "distress" state it resembles a Tragedy of the Commons with respect to time, the most limited resource of all: scientists devote weeks or months to grant writing and reviewing instead of conducting research, providing direct constructive feedback to peers, teaching, engaging publicly or advising the government.

In addition, multiple lines of evidence suggest that the idealized goals of competitive research funding systems are often unmet (OECD, 2018). Funding agencies are aware that no individual or panel possesses an inerrant ability to objectively assess “quality” or “potential,” and judgements are never totally aligned among all panel members (see also Abrams, 1991). Of course, panels do in principle aim to reach the best decisions during what is necessarily a complex and multi‐faceted evaluation process (Lamont, 2009). In our experience, effective panels will, for example, allocate much of their time to discussing applicants/applications whose initial rankings vary among panel members. Ultimately, however, the reality that all final decisions will reflect some subjectivity has been demonstrated by multiple studies (e.g., Cousens, 2019; Li & Agha, 2015; OECD, 2018; Wilsdon et al., 2015; Winder & Hodge, 2017). Subjectivity in peer review can therefore be considered an unavoidable limitation of any competitive funding system and will likely mean that there is often no difference in “quality” between research that has received funding and “the next best” (i.e., nearly funded) research (van den Besselar & Sandström, 2015). This situation might also often reflect resource limitation that results in research proposals that are evaluated by reviewers as of very high or outstanding quality but that nevertheless go unfunded. Despite its shortcomings, peer review remains the gold standard for many research communities (Wilsdon et al., 2015).

Funding decisions are also subject to increasing external influences (reviewed in Penfield, Baker, Scoble, & Wykes, 2014): pressures for societal relevance may alter panel perceptions of quality, and some researchers feel that institutional assessments like the “Research Excellence Framework” in the UK might use criteria that become ineffective surrogates for quality (Eyre‐Walker & Stoletzki, 2013). The underlying reasons for this are changes in what is viewed as good science. In many countries, excellent basic science is not on its own deemed sufficient for funding, and researchers are urged or even required to make a case for the direct relevance of their research to society (KNAW, 2018; OECD, 2018; Penfield et al., 2014). In our view, across‐the‐board emphasis on direct societal relevance is troubling: numerous historical examples highlight the serendipitous nature of scientific discovery as well as the fact that the translational impact of a particular study often occurs well after the original discovery (Gravem et al., 2017; Stephan, 2012). In addition, societal relevance can be used as a criterion for ad hoc funding decisions with a political rather than scientific basis (as, e.g., recently happened in Australia, see Nogrady, 2018). Nevertheless, there are also many positive effects of heightened focus on societal relevance, which we discuss below. One problem, in any case, is that funding agencies might not be explicit enough about the criteria and values with which proposals are being judged.

The systemic challenges facing scientific funding prompted recent calls for shifts in science funding allocation practices, such as network (Bollen, Crandall, Junk, Ding, & Börner, 2014) or lottery (Fang & Cassadeval, 2016) approaches for the distribution of funding. Vaesen and Katzav (2017) even suggested that it might be best to distribute money equally amongst all scientists without competition. We here propose that useful insights might come from comparing existing funding schemes to identify especially positive and destructive elements with respect to maintaining scientific quality and promoting efficient and positively motivated scientific communities. With this goal in mind, we leverage the substantial variation that already exists in science funding for evolutionary biology across countries to initiate a constructive, forward‐looking discussion about how funding strategy influences scientific quality in this field. Whereas a comprehensive across‐country comparison might be very difficult to achieve, even imperfect “partial” comparisons can provide important insights into the consequences of particular funding strategies (OECD, 2018; see also Laudel, 2006 and Whitley et al., 2018 for good examples).

We believe that our approach can highlight effective funding schemes and may help evolutionary biologists to identify their own “optimal niche” for funding success. Our discussions were initiated through a workshop at the European Society for Evolutionary Biology conference in 2017 that was organized by S.M. and M.N. Even though our focus is on evolutionary biology, we believe that many of our conclusions are likely to be generalizable, at least to some extent, to other scientific fields.

## 2 VARIATION ACROSS FUNDING AGENCIES AND HOW THIS VARIATION AFFECTS SCIENCE

We draw on our expertise as evolutionary biologists who collectively work in multiple countries to provide an initial sample of current practice in countries that foster major endeavours in evolutionary biology. We compare national funding schemes for evolutionary biology in ten different countries (see Table 1 for details). We also include the European Research Council (ERC), which is one major strand of the European Union's overall science funding (currently Horizon 2020). The ERC explicitly funds bottom‐up basic science and has emerged as an important funding scheme for many European evolutionary biologists. While some of us have experience on review panels or in other capacities for the surveyed agencies, it is important to emphasize that the information and views reported here are our personal impressions, compiled in late 2017 and early 2018. We also include information on newer funding schemes by the Dutch funding agency NWO, following the implementation of major changes in summer 2018. We focus our assessments and comments on 14 elements grouped into four categories: (a) topical shaping of science, (b) distribution of funds, (c) application and review procedures, and (d) incentives for mobility and diversity.

**Table 1:** Details of reviewed funding agencies. All details have been assessed in late 2017/early 2018; additional newer schemes indicated as well for the Netherlands. All details expressed are personal views

Country	Statements in Tables 2, 3, 4, 5 refer to
Australia	The Australian Research Council (ARC), the main governmental funding body for research; some of the statements are based on Discovery Projects, the ARC's main funding instrument for nonapplied research
Canada	Natural Sciences and Engineering Research Council of Canada (NSERC), Canada's federal funding agency; some of the statements are based on Discovery Grants
ERC	Mainly to the Starting, Consolidator and Advanced grant schemes of the European Research Council (ERC)
France	French National Research Agency (ANR), the main governmental funding body for research
Germany	Deutsche Forschungsgemeinschaft (DGF); more specifically, some of the statements are based on individual project grants (“Sachbeihilfe”)
Netherlands	ALW programme of the Netherlands Organisation for Scientific Research (NWO) until May 2018; since August 2018 new ENW programme (indicated where these programs differ)
Portugal	Foundation for Science and Technology (FCT)
Sweden	Swedish Research Council's board for Science and Engineering and the yearly announcement of project grants.
Switzerland	Swiss National Science Foundation (SNSF), the main governmental funding body for research. Some of the statements are based on Project Grants within Biology and Medicine
UK	Natural Environment Research Council, primarily to their “Discovery grants” and “individual fellowship” schemes. The Biotechnology and Biological Sciences Research Council operates a similar, but not identical system
United States	US National Science Foundation (NSF) and the US National Institutes of Health (NIH)

Our survey reveals some striking similarities and differences across the 11 funding agencies with respect to allocation of funding for evolutionary biology research (Tables 2, 3, 4, 5). Below, we provide our perspective on how the 14 different elements that were our focus affect scientific quality. While not all authors agree regarding all the details or even the overall thrust of each recommendation, the absence of unanimity is not surprising given that “scientific quality” is not an objectively measurable quantity on any single scale. Nevertheless, we hope that our discussion can inspire a constructive debate amongst both researchers and funding agencies on important issues surrounding mechanisms of science funding allocation, scientific quality and the health of the scientific community (see here also Cousens, 2019).

**Table 2:** Assessment of elements regarding the topical shaping of science across funding agencies

Country	Emphasis on societal relevance/broader impacts?	Investment in bottom‐up blue‐sky research vs. top‐down funding programmes	Integration of funding programmes for basic and applied science/science with societal relevance?
Australia	Yes	Mostly bottom‐up	Same funding agency but different instruments; societal relevance also important for basic science projects
Canada	Yes	Mostly bottom‐up	Yes
ERC	No: Emphasis on scientific excellence but societal relevance is considered	Exclusively bottom‐up	Yes: Main schemes fund basic research but supplementary schemes are available to develop impact
France	Yes: Projects focusing on 9 major societal challenges (50% of funding)	Half bottom‐up/half top‐down	Yes: Same funding agency, some calls offer possibility to integrate both types of projects
Germany	No: Emphasis on basic research, but follow‐up “transfer” funding with nonacademic partners possible	Mostly bottom‐up; ~7% of DFG funding goes into top‐down “Priority programmes”	Same funding agency but different instruments, for example clinical trial grants
Netherlands	Yes: Societal relevance 20% of the total score (ALW); this has recently changed to impact and risen to 30% in some calls (ENW)	Bottom‐up; some specific calls; consortia often have specific constraints	Yes: Societal relevance and/or impact important. ENW: Some consortia require industrial or societal partners who co‐fund the project; consortia topics can be informed by societal/economic relevance (Top sectors; Dutch national research agenda)
Portugal	Yes: Very important	Bottom‐up	Yes: same funding agency, same scheme
Sweden	No: Only scientific value is considered.	All bottom‐up; but also some specific calls	No: There are other governmental funding bodies that announce grants with societal relevance
Switzerland	No: Emphasis on high‐quality basic research	Mostly bottom‐up	No: Basic research funded by SNF, applied science funded by KTI, which is done at technical colleges
UK	Yes: Societal impact is considered in all funding schemes, including “Discovery grants” (funding route for basic science)	1/3rd bottom‐up; also some scientific community input to strategic research programmes	Yes: All schemes require some contribution to societal impact; the extent of the contribution required varies among schemes
United States	Yes: Broader impacts required for NSF; NIH grants relevant to human health	Mostly bottom‐up	Same funding agency, increasing emphasis on funding for broader impacts activities within basic science grants at NSF.

**Table 3:** Assessment of elements regarding the distribution of funds across eleven funding agencies

Country	Allocation of money	Consortia vs. personal stipends	Long‐ vs. short‐term	Flexibility of funding schemes	Funding rates, and who can apply?
Australia	Mostly intermediate (~400 k$), but also a few large grants	Project grants predominate; teams with >1 applicants normal	3–5 years for research projects	Flexible (budget between $30 k and $500 k per year)	Funding rate: ~18% (Discovery Projects); all employees of Australian Universities can apply, and there can be international partner investigators. Max of two grants per person
Canada	Small amounts (35 k$ typical), many awardees	Personal stipends	5 years	Flexible	Funding rate: 70%–75%. Only Prof. or Adjunct Prof. can apply
ERC	Large (1.5–2.5 M€)	Mostly individual‐led projects; synergy grants for cross‐disciplinary teams	5 years	Flexible, but ambitious projects expected	Funding rate: ~10%. Restrictions on working time since PhD (Starting and Consolidator). Applicants must have a base in a suitable EU institution.
France	Intermediate to large (200–900 k€)	Consortia grants predominate; 75% collaborative projects	2–4 years	Flexible	Funding rate: 10%–15%. Permanent researchers at University/Research Center can apply
Germany	Intermediate (~230 k€); larger grants for consortia etc.	Project grants with single applicant predominate	≦3 years for research projects	Flexible	Funding rate: 36% (Individual research projects). Researchers holding a PhD at all German research institutions can apply
Netherlands	ALW: intermediate; ENW: various types, from small to large (160 k€–3 M€), some even bigger grants	ALW: Personal grant to PI ENW: Personal grant to PI, personal grant with co‐PI or large (consortium)	ALW: 4 years	ALW: Quite fixed ENW: relatively flexible for bigger ones	Funding rate ~10% (data for ALW; no data yet for ENW). Permanent faculty can apply, tenure‐track PIs with declaration that the applicant will be hired for project duration
Portugal	Intermediate (up to 200 k€)	Both. Not clear which is preferred	3 years	Quite fixed; budget justifications	Funding rate between 5% and 8%. Anyone with a PhD is allowed to apply
Sweden	Intermediate (400 k€) and many awardees	Project grants to main applicant	4 years	Very flexible	Funding rate 20%. Staff affiliated to Swedish university at least 20% of their time can apply
Switzerland	Intermediate: regular grants (~500 kCHF); also some larger grants	Project grants predominate, single applicant preferred	1–4 years for research projects	Flexible (budget of at least 50 kCHF)	Funding rate: 43% (Project grants in Biology & Medicine). Researchers ≥4 years post‐PhD. Only one application per round, up to two in total
UK	Intermediate: 65‐800 k£; also large grants	Project grants predominate, often teams. Fellowships also available.	3 years for projects, 5 for fellowships	Flexible, but detailed cost justification required	Funding rate: ~20%. Only researchers with contracts extending beyond the grant period may apply. Institutional application quotas apply
United States	Intermediate (~100–250 k$); also some smaller and bigger grants	Project grants predominate, teams with >1 applicants are common	Typically 2–5 years	Somewhat flexible; NSF requires budget justification	Funding rate: <10%–25%. Who can apply is dependent on the grant (often PI status needed, some to post‐docs and graduate students)

**Table 4:** Assessment of elements regarding the application and review procedures across funding agencies

Country	Who/what is being judged	Administrative burden/length of proposals	Who is reviewing?	Existence of interviews and rebuttals
Australia	Main scheme: 40% project quality, 35% investigators, 10% feasibility, 15% benefit	High burden: In total, an application >50 pages, often several investigators	Panel of experts, external reviewers	Rebuttals
Canada	Excellence of candidate, proposal quality, Highly Qualified Personnel Training	Intermediate burden: 5 pages or research proposal plus budget, HQP training and CV	Panel of experts, external reviewers	No rebuttal, no interview
ERC	Project and investigator; over‐riding criterion is scientific excellence	High burden: Round 1 (5‐page proposal + CV, track record) and Round 2 (20‐page proposal, budget proposals) submitted together	Round 1: Panel, round 2: panel and external reviewers	No rebuttals, interview at Starter or Consolidator level
France	Quality and originality of project, quality and expertise of consortium, adequacy of budget, impact and diffusion strategy	High burden: Round 1 = project of 4 pages and CVs. Round 2 = project of 20 pages and CVs	Panel of experts, external reviewers	Rebuttals since 2016, no interview
Germany	Scientific quality, applicants’ qualifications, objectives and work programme, employment opportunities, planned allocation of funding	Intermediate burden: 20 pages maximum for research proposal, plus CV	Panel of experts, external reviewers	No rebuttals/interviews for project grants
Netherlands	Originality of proposal, scientific quality (proposal and team), societal relevance and/or impact	ALW: Relatively low burden: total proposal 12 pages; ENW: around 8 pages for research proposal	Panel of experts, external reviewers	2‐page rebuttal. Some schemes: interviews
Portugal	Project, team and investigator	High burden	Panel and external reviewers	Rebuttals; no interview
Sweden	Novelty and originality, scientific quality and merits of main applicant.	Low burden: Project description max 10 pages. Budget uses a template. Reuse of CV in system	Panel of experts	None
Switzerland	Track record, scientific quality and feasibility	Intermediate burden: Research plan 20 pages, CV = 2 pages, list of achievements = 2 pages	Panel of experts, external reviewers	None for project grants. Fellowships: Interviews
UK	Scientific quality of the project is main criterion; also investigator track records, risk‐reward balance and impact	Intermediate burden: 8‐page proposal plus budget, CVs, Impact statement and forms. Internal vetting before submission adds burden	Panel of experts, input from external reviewers	Rebuttals considered, interview for fellowships
United States	Scientific quality, applicant qualifications, diversity, impact, programme portfolio	High burden: 12‐ to 15‐page project description along with many supplementary documents	Panel + reviewers (NSF); NIH: Panel	None

**Table 5:** Assessment of elements regarding incentives for mobility and diversity across funding agencies

Country	Mobility	Focus on diversity, equal opportunities
Australia	Not emphasized	Accounted for to some extent through “performance relative to opportunity” assessment
Canada	Not emphasized	Explicit focus on equal opportunities
ERC	Emphasized at Starting Grant level	Explicit focus on equal opportunities
France	Not emphasized	No specific focus on equal opportunities
Germany	Emphasized only for post‐docs	To some extent: Diversity and equal opportunity are recognized as important; special benefits for fellowship recipients with children
Netherlands	Emphasized only for post‐docs	Not focused upon in ALW‐scheme, but women prioritized in ENW scheme. For excellence schemes: extensions for eligibility period for parenthood after doctorate (18 months of standard extension per birth for women, up to 3 children; also extension for documented care‐taking time for fathers); special NWO grants for women outside of ALW/ENW
Portugal	Emphasized for fellowships	No focus on equal opportunities
Sweden	Emphasized only for post‐docs	Explicit focus on equal opportunities
Switzerland	Emphasized only for post‐docs	Special grant for female researchers with family‐related career interruptions; extensions of eligibility periods for excellence scheme (Ambizione) in case of maternity after doctorate (18 months per child or longer if documented)
UK	Emphasized for fellowships	Explicit focus on equal opportunities
United States	Not emphasized	Explicit focus on equal opportunities

## 3 TOPICAL SHAPING OF SCIENCE

### 3.1 Emphasis on societal relevance and broader impacts

Some of the national funding agencies that we review prefer, or even require, that basic science projects have societal relevance (e.g., Australia, Canada, France, Portugal, UK, United States, the Netherlands; Table 2). Our survey suggests that the way in which societal relevance is implemented differs across funding agencies (see also OECD, 2018). For example, some countries (e.g., UK, United States) merely require some form of representation or translation of basic science to the public and/or policy arenas, whereas other countries have a more direct requirement for science with a societal value, often to the potential detriment of basic science (e.g., Portugal, France, the Netherlands). By contrast, there is no specific requirement for the inclusion of societal impact for national funding agencies in Switzerland, Germany, Sweden or in the ERC. Indeed, part of the motivation underlying the establishment of the ERC was as a counter to the increasing emphasis on societal relevance in other EU funding instruments (currently Horizon 2020, see also Nowotny, 2006).

Whether the strategy of explicitly requiring societal relevance leads to better (broadly conceived) science is an open question. In general, it is very difficult to measure broader impact (KNAW, 2018; LERU, 2018; Penfield et al., 2014; Wilsdon et al., 2015). Not surprisingly, we as a group are somewhat divided regarding this issue. In the best case scenario, a societal relevance requirement would improve scientific and societal progress (Rinze & Miedema, 2016). In particular, encouraging scientists to take a broader view of values that include societal impacts might liberate them from the potentially harmful yet sometimes still entrenched stance that science is best conducted in a cultural, social and historical vacuum. Good examples of where evolutionary biology can have policy implications and thus usefully addresses “science in society” come from research programmes directed at understanding how anthropogenic influences affect natural population dynamics and evolution in urban settings (Alberti, Marzluff, & Hunt, 2017; Alberti, Correa, et al., 2017), in response to climate change, hunting, agriculture, pollution, or antibiotics (Hendry, Gotanda, & Svensson, 2017) or integrating evolutionary understanding of underlying processes with ecological monitoring of biodiversity loss (Brodersen & Seehausen, 2014).

On the flip side, scientific quality might suffer from an increased focus on societal relevance if researchers abandon the most important questions or problems in an effort to address short‐term issues that fit current policies or agendas (KNAW, 2018). Often, these projects or calls for proposals focus on delivering economic or technological pay‐offs (Gibson & Hazelkorn, 2017) and seem motivated by the need to account for taxpayer contributions to national science funding. A related but distinct problem is that the extreme competition that characterizes many grant programmes might incentivize researchers to exaggerate potential societal benefits (so‐called “grant‐speak”). In our view, this latter issue is especially likely in situations where funding outcomes directly depend on the perceived societal relevance of the expected short‐term project results. It would thus be a distinct improvement if grant proposal evaluations focused less on expected project outcomes and more on project design. Such focus on methods, or “pathways to impact” (KNAW, 2018; LERU, 2018), can, for example, include evaluation components that address whether projects involve stakeholders or include real‐world input such as field research (wherever appropriate). In our view, funding agencies should ideally also maintain substantial funding for basic research per se.

### 3.2 Investment in top‐down funding programmes versus bottom‐up blue‐sky research

National funding agencies typically offer both bottom‐up and top‐down funding programmes, but our survey suggests that there is variation across these agencies in the proportion of investment in each type of programme. Top‐down funding streams are directed towards specific goals and purposes, often with a societal, technological or economic focus (see also section above). Our overview (Table 2) suggests that the UK has the largest share of such top‐down programmes for evolutionary biologists, around two‐thirds of the funding programmes that support evolutionary biology in the UK. Such relatively heavy investment in top‐down funding is also linked to the fact that some new UK funding streams are now available in the specific context of the Official Development Assistance (ODA) fund. Around half of France's funding programmes for evolutionary biology are invested in a top‐down context. The other funding agencies primarily offer mostly (or only, ERC) bottom‐up blue‐sky funding for evolutionary biology, though funding in the Netherlands can also come with specific constraints and/or in a top‐down context.

Whereas top‐down research funding streams are typically directed to the most pressing needs of a specific society, a top‐down focus also increases the likelihood that researchers are faced with a relatively narrow set of perspectives and possibilities. By contrast, in a blue‐sky system, researchers can freely choose topics and methods. Whereas this latter approach might be viewed as risky, the typical top‐down pathway of following current trends and hypes can prove suboptimal: “big ideas” can fail to deliver what they promised, generating substantial long‐term risks via heavy investment in an ultimate failure (Joyner, Paneth, & Ioannidis, 2016). There also exists the substantial concern that (too) many researchers working on the same topic might promote incremental thinking while decreasing the likelihood of breakthroughs in unexpected directions (Geman & Geman, 2016). We believe that it is thus especially important to preserve and even increase investment in bottom‐up “blue‐sky” funding schemes because, in our view, these strategies provide a funding mechanism that is more likely to be associated with high‐quality research and that could also provide substantial societal benefits via connections to relevant stakeholders. Indeed, this could be tested: a recent bibliometric study demonstrated that breakthrough‐type (“disruptive”) research has not typically been the type of research that had been funded by the US NSF (Wu, Wang, & Evans, 2019). It would be interesting to investigate whether, for example, the ERC as an entirely bottom‐up funding scheme does deliver this type of science.

### 3.3 Integration of funding programmes for basic and applied science and science with societal relevance

Funding agencies in some countries score grant proposals by integrating separate scores for basic and applied components of the proposed research (e.g., United States, the Netherlands), whereas others evaluate applied and basic aspects together (Portugal, Australia; see also Table 2). A different model is provided by countries in which basic and applied research proposals form separate funding streams, handled by different funding agencies (e.g., in Switzerland) or committees (e.g., Discovery grants versus Strategies grants in the Canadian NSERC; Swedish Research Council (Vetenskapsrådet) versus the Swedish Research Council for Environment, Agricultural Sciences and Spatial Planning (Formas); Societal challenge axes versus fundamental axes in the French ANR). The ERC funds basic research, but offers supplementary schemes for subsequent development of societal–technological impact.

These different approaches can have major consequences for the types of projects that are funded. In theory, funding agencies or programmes that handle both basic and applied science can enable projects to bridge the basic‐applied divide. In practice, this scenario can lead to a situation where basic and applied science proposals are placed in direct competition, often to the detriment of fundamental science. Such competition is reduced when separate funding schemes are used for basic and applied science, though this separation might generate new challenges. First, specific types of funding might become tied to certain institutions, making it difficult for researchers from other institutions to obtain this type of funding even if their research is applicable. Second, there is the top‐down issue of how much money flows into each pot. In our view, funding agencies should provide some funding dedicated to basic science because this strategy can ensure that basic science always receives support. This reasoning also takes into consideration that applied science projects are more likely to be suitable for funding or co‐funding sources that exist outside of national funding agencies, such as private sector end‐users.

## 4 DISTRIBUTIONS OF FUNDS

### 4.1 Allocation of money: Large versus small grants

How money allocated to research grants is distributed differs substantially across the funding agencies. Our survey suggests that Canada provides low levels of funding per grant relative to the intermediate‐level grants typical of other countries in our survey and the relatively large grants provided by the ERC and recently, the Dutch NWO (see Table 3). We also find that grant sizes vary within funding agencies.

In principle, relatively large grants might be preferable in situations where the technology required to achieve particular desired outcomes is very expensive or when a few researchers do such an outstanding job (judged by past performance) that exceptional results are also expected for future work. The latter argument stands on shaky ground; however, more funding does not necessarily imply higher scientific output—or at least not to the degree expected (Fortin & Currie, 2013). In our view, the only argument for high investment in a few projects that seems to withstand scrutiny is that breakthrough research might require a great deal of financial investment. Scientific breakthroughs often occur via outside‐the‐box thinking, which can be enhanced when a diverse team of researchers works together to solve a specific scientific question or problem (Bammer, 2017; Bromham, Dinnage, & Hua, 2016). For this reason, the funding strategies directed towards so‐called excellence centres often involve relatively large pots of money that are granted over substantial periods of time (Bloch & Sørensen, 2015).

On the other hand, we believe that there are several objective reasons to favour a more egalitarian distribution of grant funding. In particular, truly “breakthrough” research is very rare and still needs a foundation provided by “normal” research projects. It is also reasonable to consider that a more egalitarian distribution of resources might translate into a happier scientific community that might in turn produce better science and reduce the incidence of fraud (Moore et al., 2017). The “happiness” point finds indirect support from research on the determinants of societal happiness, which indicates that the lack of fundamental resources or rights can generate marked unhappiness. With respect to fraud, resource scarcity has been implicated as contributing to the incentivization of fraudulent fabrication or omission of data, result enhancement, idea stealing and monopolization of critical resources (at least in the United States; Anderson, Ronning, De Vries, & Martinson, 2007). On the other hand, because administrative burden might scale at least in part with the number of funded grants, a major increase in the funding rate associated with a decreased allocation of money per grant does not come entirely cost‐free. Overall, we believe that a targeted funding strategy that allocates most funds towards a broad base (i.e., smaller individual awards) but also includes a few larger awards (for large interdisciplinary projects and/or centres of excellence) might be the best way to increase overall systemic quality. Relatively large project funding is typically awarded to relatively large teams (consortia or centres) rather than individuals (though there are exceptions, e.g., ERC grants).

### 4.2 Consortia versus individual‐led projects

Some funding agencies include schemes that give money to relatively large teams of researchers (so‐called “consortia”; see also Bloch & Sørensen, 2015). Consortium‐based approaches are fundamentally different from individual‐led projects, where funding is primarily awarded to individual researchers with excellent ideas and/or with a track record of excellence. Such a separation between funding strategies for consortia and individuals does not mean that a funding agency cannot support both types of project (e.g., Horizon 2020).

Our overview suggests that consortia‐based funding schemes are relatively rare in evolutionary biology. France is an exception, where 75% of grants are awarded to consortia. Relatively large consortia grants are also awarded in the Netherlands and in some UK schemes. Whereas several of the other countries provide grants to more than one PI (Portugal, Australia, United States; Table 3), none of these countries typically offer funding schemes that focus on large‐scale consortia. Several countries do provide funding for “centres of excellence” or larger network schemes (e.g., Australia, Switzerland, Norway, Sweden and Finland), which can include evolutionary biology.

Individual‐led programmes, which provide the largest share of grant types in our overview, can be objectively split into two different categories. First, for so‐called “project grants,” funding is given to established researchers whose baseline salary is often covered by their employing institution but can sometimes also be partly cost recovered in the grant. These grants typically include funding for junior researchers (i.e., post‐doctoral researchers and/or PhD students). The other type of individual‐led programs, offered by several countries in our survey, provides personal research fellowships. These awards tend to be allocated to relatively recent PhD recipients (e.g., Switzerland, Germany, UK). Australia and the Netherlands offer three different fellowship schemes, for independent junior, mid‐career and senior researchers.

Both large team and individual‐led projects offer advantages and disadvantages. On one hand, consortia and centres can yield benefits by integrating a variety of perspectives (Bammer, 2017; Ledford, 2015). These groups also typically consist of established scientists who are relatively likely to produce high‐quality science (Bloch & Sørensen, 2015). On the other hand, larger groups can also stifle creativity and tend towards conservatism (Geman & Geman, 2016), and may actually waste resources if scientifically unnecessary partners are included solely to fulfil funding criteria. The flexibility and freedom of choice and methods that characterize relatively small‐scale projects can produce surprising scientific outcomes that are not likely to be generated by consortia or other large groups (Wu et al., 2019). By this logic, we believe that maximizing scientific quality will include a balanced investment in both individual projects and group‐led efforts (see also Wu et al., 2019).

### 4.3 Long‐term versus short‐term projects

Our survey shows that funding is most often associated with 2‐ to 4‐year projects, with the exception of fellowships (see Table 3; see also OECD, 2018: clustering found around 3‐ to 5‐year funding). In many countries, grant duration is likely tied to the duration of typical PhD and post‐doc positions, which can vary widely across countries. There are relatively few funding agencies and schemes that support longer‐term projects (e.g., NSERC [Canada], ERC, some NSF funding schemes, “centres of excellence”).

How does the typical focus on relatively short time scales influence scientific quality? In our own field of evolutionary biology, it is impossible to generate robust insights into many fundamental questions (e.g., natural temporal fluctuations in selection) within short time frames (Clutton‐Brock & Sheldon, 2010). Indeed, the longest running field studies in evolutionary ecology are some of the most productive (Clutton‐Brock & Sheldon, 2010; in particular their box 3), and there is a growing consensus that long‐term research in ecology and evolution offers unique and important insights (Hughes et al., 2017; Kuebbing et al., 2018). Despite the clear value of long‐term studies, there is a real concern that funding schemes will push biological research away from long‐term field‐based work in natural populations towards laboratory‐based research with model organisms, simply because the time frame and feasibility of laboratory‐based research provides a better fit to current funding schemes (see also Kuebbing et al., 2018; Neiman, Meirmans, Schwander, & Meirmans, 2018). Clearly, funding strategies need to support both types of research, ideally working together.

We believe that the documented productivity and quality of long‐term research projects should counter the viewpoint that this type of project is “too risky” in drawing resources for a long period of time without producing tangible benefits. For this reason, we suggest a stronger emphasis on more long‐term funding as implemented by some funding agencies (see also Alberts et al., 2014). Even so, we recognize the challenges associated with using a finite pot of money to manage the trade‐off of providing long‐term projects with guaranteed funding while simultaneously encouraging diversity in research groups, including support for small and recently established (or to be established) research teams. Monitoring long‐term research and/or developing a low‐burden application process for continuation of especially promising and already funded projects (e.g., Switzerland) seems, in our opinion, to constitute a move in the right direction.

### 4.4 Flexibility of funding schemes

Most funding agencies show some flexibility at least in principle regarding project duration and budget (Table 3). However, in our experience the reality can also be that scientists may need to show sufficient ambition, meaning that the maximum funding possible needs to be requested for in order to be assessed at all (e.g., the Dutch excellence schemes). Good arguments can probably be made for inflexible approaches and particularly from the managerial perspective of funding agencies. Nevertheless, we believe that existing flexibility should be enhanced by allowing each funding scheme to be tailored to the actual needs of any specific project—be it budget, duration or other aspects. For example, whereas some projects could be reasonably well equipped with relatively little money (e.g., modelling, meta‐analysis, reviews), other projects will need larger sums of money and/or time just to get started (money: projects using genomics; time: e.g., studies on senescence, Monaghan, Charmantier, Nussey, & Ricklefs, 2008). Thus, it seems that more flexibility could substantially boost both quality and relevance of the funded research.

### 4.5 Funding rates and who can apply

Our survey shows that funding rates for evolutionary biology research vary dramatically within and across countries, ranging from 5% to 75% (Table 3; see also OECD, 2018). This variation in funding rate is especially interesting from the perspective of the idea of a “tipping point”: funding rates lower than 25%–30% might drive the system towards a “distress” state (Edwards & Roy, 2017; see introductory text), suggesting that distress might characterize several of the countries that we surveyed.

Our survey also highlights mechanisms underlying high funding rates in certain countries or schemes, and in particular points to the roles of different demand management schemes: in essence, policies that restrict applications increase funding rates. Thus, we suggest that, if properly applied, demand management schemes might decrease the risk of the “distress state.” Accordingly, in our opinion, funding agencies should carefully consider the application of some variant of these schemes. For example, several countries restrict applications to researchers with permanent positions (in addition, whereas these types of grants cover costs for PhD students and materials, they do not cover the applicant's salary, and personnel costs typically translate into a relatively high cost for a given proposal). Another possibility is demonstrated by agencies like the Swiss SNSF that do not allow researchers to hold more than one grant on similar topics. Yet another potentially useful mechanism to manage demand is provided by agencies that limit researchers to a certain number of applications to a particular funding scheme (e.g., Veni and Vidi grants in the Netherlands, though funding rates still remain very low for these grants).

In the UK, the Natural Environment Research Council (NERC) sets university‐level quotas for applications that are based on the previous number and success of applications: only those universities that have already produced excellent work can submit relatively high numbers of applications. Although this quota strategy maintains the NERC funding rate at a higher level (~20%) than it would otherwise be, the imposition of a quota produces an earlier within‐university selection process. We as a group have mixed experience with such quotas: on the one hand, they can seem unfair to an individual, but on the other hand, quotas can promote collegiality in the application process and more effective prescreening of proposals in a group. Indeed, quotas can reduce the tail of low‐quality proposals, including proposals submitted for the sole purpose of submission per se, a frequent outcome of the increasingly common requirement of proposal submission laid out in some university contracts.

The ERC is unique in attempting to increase funding rates by setting the bar high at the proposal stage: only the most “excellent” researchers are encouraged to apply, and individual applicants whose proposals go unfunded are not allowed to reapply for up to two years if the proposal is deemed too far below the threshold for funding. A preproposal requirement (e.g., US NSF, though this requirement was recently dropped; French ANR) is another mechanism that can be applied to increase funding rates at the full proposal level. Whereas all of these demand management schemes have limitations, thoughtful application of such schemes does seem to have some promise for increasing funding rates in a way that minimizes wasted applicant and reviewer time.

One of the most obvious challenges associated with demand management schemes is the attendant risks of enhancing the “Matthew effect” (Merton, 1968), defined as a situation where researchers who are already successful receive, for each unit of work or “quality” they produce, more credit than relatively new or junior scientists. Demand management schemes might also decrease the likelihood that vulnerable researchers (minorities, women, individuals following alternative career pathways) will achieve funding success. This latter issue is particularly problematic with respect to, for example, the notable and thus‐far intractable gender gap in science (Holman, Stuart‐Fox, & Hauser, 2018). In our view, this issue could be ameliorated by earmarking funds for underrepresented and vulnerable groups (see section on diversity and equal opportunities below). One might also consider circumstances that affect performance scores. A good example of such consideration is provided by the Research Opportunity and Performance Evidence (ROPE) Statement incorporated in the Australian funding scheme. This statement allows researchers to explain career histories and opportunities, providing an opportunity to explain, for example, career breaks due to family obligations.

Finally, we believe that there should be limits imposed on the rigidity of management schemes. For example, management schemes that prevent resubmission (e.g., BBSRC in the UK) might ultimately reduce quality by generating barriers to revision and reconsideration of promising proposals.

## 5 APPLICATION AND REVIEW PROCEDURES

### 5.1 Who/what is being judged?

Our overview shows that applications are judged similarly across our surveyed funding agencies (Table 3): all of the evaluation processes focus on both project and researcher/team quality, in accordance with the core missions of funding agencies (see also OECD, 2018). We also did find some across‐country differences in evaluation criteria, such as an added focus on societal relevance/ impact. Australia also applies specific weighting percentages across the different criteria for judging proposals. The US NSF has an additional focus on programme portfolio, meaning that this agency considers criteria that ensure the overall diversity of the research they support (e.g., balance across universities, geographic regions, disciplines and approaches). In our view, the NSF portfolio approach thus likely enables a good balance of support and minimizes the negative influence of (over)enthusiasm for research trends, with the cautionary note that portfolio definitions are themselves subject to trends and political influences.

We can consider the advantages and disadvantages of the apparent across‐country variation in proposal evaluation criteria and whether there could be better ways to structure the evaluation process. A focus on researcher quality might free scientists to focus on relatively risky projects, whereas emphasis on project quality could provide a foot in the door for researchers with a career gap, from alternative career pathways or from a different area of expertise. Team quality enhances project quality via complementary expertise but can also restrict funding to those researchers with well‐established scientific networks. We believe that it might be especially worthwhile to consider whether different criteria should be applied at different career stages or to different types of projects. For example, one could imagine a focus on researcher quality primarily for more established researchers in a permanent position, while giving preference to project quality for younger researchers on temporary contracts.

### 5.2 Administrative burden/length of proposals

We found that grant proposals vary substantially across funding agencies in length and structure, the number (if any) and type of support letters that are required, and the extent of budgetary detail needed (see Table 4). Whereas such variation might seem mundane or unimportant compared to variation connected more obviously to science, such details can dramatically influence the amount of time and energy needed to write and submit a proposal, and the review process. Proposal structure will also have a major impact on the administrative burden imposed by submission and processing.

In principle, shorter and/or simpler proposals should impose a relatively light burden on scientists, reviewers and administrators, and we therefore strongly advocate changes in this direction. Shorter and simpler proposals will free up time and resources for science itself and might be relatively easy to achieve. Indeed, this logic is a major justification for the employment of preproposals by some funding agencies (e.g., until the last year by the US NSF). The extent to which preproposals reduce peer‐review burden remains unclear, however. For instance, the implementation of a preproposal requirement for French ANR grants in 2014 resulted in a >50% increase in grant applications. The ERC, on the other hand, requires simultaneous submission of long and short versions of the grant application, which seems to work well on the panel/reviewer side without generating very large numbers of preproposals. However, the ERC model does impose a substantial burden on applicants, who might invest substantial time in writing a proposal that goes unread and thus, yields no feedback. For this reason, we believe that a focus on a single short and simple proposal is likely to constitute a substantial improvement (around eight pages for the project description). One could also consider whether it would be helpful to tailor grant length more specifically to the evaluations needed (see section above): when judging primarily for researcher quality (track record), the application could involve a long CV and a short project proposal, whereas project quality‐based grant programmes could require a relatively long project proposal and a short narrative CV that focuses more broadly on achievements.

### 5.3 Who is reviewing?

Most of the eleven reviewed funding agencies use a combination of panel‐based reviews and/or external peer reviewers to evaluate proposals (Table 4). The two exceptions in our table are the US NIH and Sweden, which rely exclusively upon panel reviews. There is also variation in the extent that panel compositions use, or exclude, scientists who work in the country whose applicants are being judged.

How does variation in peer‐review strategy affect scientific quality? The standard view is that only external peer reviewers have the specialized knowledge needed to truly judge the quality of a grant application; grant review panel members do not necessarily possess specialized expertise for a particular proposal. There is also the distinct potential for a negative “inbreeding” effect (i.e., nepotism) that can result from panels comprised of reviewers from the same country. Extensive use of international peer review (or even an international review panel, e.g., Finland; see also OECD, 2018) is one way to minimize any potential effects of nepotism. This perspective thus implies that the sole use of internal panels for reviewing applications is a less‐than‐optimal choice, at least if this panel is largely comprised of reviewers from that particular country. The reality is, however, that it is increasingly difficult to find enough senior expert peer reviewers to review a growing proposal load, meaning that many such “experts” might in reality often be relatively junior scientists (see also Alberts et al., 2014). These junior scientists may lack sufficient breadth of experience of science and the funding context to evaluate diverse proposals; on the other hand, they also might invest more time and perhaps perform a more thorough review than more experienced scientists. There is also a reasonable concern that review confined to older experts might translate into narrow perspectives that discourage outside‐the‐box thinking. Both more junior scientists and panels might benefit from broader and more diverse perspectives, including the possibility to discuss the proposals amongst panel members (see Lamont, 2009).

This is the basis for our conclusion that a combination of panel and external reviewers, as now used by most funding agencies, might indeed lead to the best and fairest possible outcomes. It is also important to reduce the impact of nepotism via international panels and peer reviewers–this consideration is likely the more important in relatively small countries with relatively few scientists. We do want to emphasize one important caveat: even within a panel, excellent but risky proposals or integrative/interdisciplinary proposals might have relatively low chances of success, at least if funding itself is rigidly focused on research feasibility or is structured into funding for specific research fields. Several studies have shown that whereas interdisciplinary projects are often encouraged in principle, they might be disadvantaged in practice because grant review itself has remained largely monodisciplinary (Bromham et al., 2016; Kwon, Solomon, Youtie, & Porter, 2017). Such an approach to review is likely to disproportionately affect interdisciplinary projects for several reasons, including the challenges inherent in convincing a narrow review board that a proposal integrating across concepts and/or methods is interesting and feasible and that the researchers possess necessary expertise. An obvious fix is that the review process itself becomes more explicitly interdisciplinary (e.g., review across panels).

### 5.4 Existence of interviews and rebuttals

There is considerable variation in whether funding agencies include interviews and/or rebuttals in their application process (Table 4). Rebuttals are short written responses by the applicant to the reviewer assessments, enabling the researcher to clarify issues and explain misconceptions. In interviews, applicants can also do so; in addition, panel members are here given an opportunity to “separate the wheat from the chaff.” In particular, asking for more detail can flag grand‐sounding but ultimately weak application components. One real challenge posed by interviews is the potential for bias linked to researcher characteristics (e.g., gender, ethnicity) or personality (e.g., favouring more extroverted candidates). Either way, the addition of interview or rebuttal components increases the peer review and applicant preparation load. Interviews can also be costly if they require more time and travel; the latter also generates environmental impact that could perhaps be avoided.

Only a few funding agencies currently use rebuttals (e.g., Dutch NWO; NERC and BBSRC in the UK, French ANR, see also Table 4). We believe that a well‐executed rebuttal system provides a powerful means of applying proposal review in a manner that can increase the quality of funded research (in particular research design). In our opinion, the positive features of rebuttals seem to outweigh its negative effects, especially if the rebuttal is short (e.g., two pages in the Netherlands). In particular, rebuttals could be an important alternative in cases where interviews require relatively long travel periods. Rebuttals also allow more considered answers, which might permit the researchers to provide a higher‐quality response to reviewer critiques than an interview might deliver.

## 6 INCENTIVES FOR MOBILITY AND DIVERSITY

### 6.1 Mobility

Whereas some funding agencies provide fellowships that cannot be used in the home country (e.g., Switzerland, Germany, Sweden, the Netherlands), thus favouring mobility, other funding agencies exclusively fund within‐country fellowships (United States, France, Australia; see Table 5). Appropriate mobility likely has positive effects on scientific quality: breakthrough science is often associated with transcendence of disciplinary or cultural horizons, both of which can be enhanced through international experience. By this logic, many might argue that scientific breakthroughs require novel interactions. Even with respect to the healthy progress of “normal” science (Kuhn, 1962), many scientists and science managers believe that young researchers should build their career in settings other than that of their PhD supervisor. Indeed, several recent studies detected a positive relationship between scientific quality and the mobility of scientists within a specific country (Adams, 2013; Sugimoto et al., 2017; Wagner & Jonkers, 2017). The take‐home message is that, from a scientific perspective, it thus seems very worthwhile to motivate mobility.

Despite these overall advantages, however, an emphasis on movement also poses challenges with respect to equity. Some individuals are more able to move, both physically and thematically, than others. A reluctance to move might have strong justifications—for example, a well‐established field project with a high likelihood of success associated with continued investment, or a low likelihood of employment for scientists with a working partner or family ties/dependants (e.g., school‐age children, elderly parents). Both could pose such challenges to mobility that scientific quality might effectively decrease. We therefore suggest that some mobility funding structures should become more flexible, enabling a more individual account of the project and researcher in question. Alternatively, funding schemes could also support short‐term visits across research groups, providing incentives for international collaborations per se (e.g., “mobility” credit for cross‐nationally multi‐authored articles), and even reconsider the notion that international collaboration for younger scholars and researchers necessarily needs to involve an extended tenure in a different country.

### 6.2 Diversity and equal opportunities

Some programmes or funding agencies explicitly incorporate strategies aimed at increasing diversity and the inclusion of particular underrepresented groups (see Table 5). For example, there is a Discovery Indigenous programme in Australia, and some Swiss funds are reserved for eastern Europeans (PROMYS) and for female researchers (PRIMA). There are also funds earmarked for female researchers in the Netherlands (Aspasia, Athena). In the UK and at the EU level, there are funds dedicated to enabling re‐entry into science after a career break (Wellcome Trust's Research Career Re‐entry Fellowship; Marie Curie Reintegration Grant) as well as funds to facilitate managing both family and work (Dorothy Hodgkin fellowships). Several funding agencies also take parental leave into account when determining eligibility for particular funding programmes aimed at junior researchers (e.g., Netherlands, ERC, Norway) or provide an opportunity to explain career development gaps in grant applications (ROPE statement, Australia).

These examples of diversity and equity‐focused initiatives are still relatively scarce. We believe that a continued increase in explicit consideration for diversity and equality remains important, especially in light of the multiple studies suggesting that the likelihood of breakthrough research increases when a research team harbours a diversity of researchers (Freeman & Huang, 2014; Powell, 2018). Importantly, such enhancement of the potential for breakthrough research only occurs for processes of true integration of perspectives and not for “representational” diversity (Smith‐Doerr, Alegria, & Sacco, 2017). We thus believe that funding agencies should increase their investment in funding support that is structured to maximize true integration of perspectives. The latter presupposes appropriate consideration for how details of research organization affect such integration (e.g., ensure that not all female researchers are junior researchers whereas male researchers are senior).

## 7 SOME RECOMMENDATIONS FOR BEST PRACTICES

Evolutionary biology and other scientific disciplines face the positive problem that there are more excellent researchers, with more excellent research ideas, than can be funded with available research resources. Because it seems unlikely that the available (national/governmental) resources will increase radically and/or that the pool of eligible researchers will shrink drastically in the near future, the research community and associated funding agencies have the responsibility to find the “best” way of allocating limited resources to achieve the greatest returns in terms of generating excellent science and an effective, engaged and maximally productive scientific community. In this paper, we have presented a synthesis of a constructive and illuminating discussion and co‐authorship process regarding which current elements of funding allocation might prove most useful. These discussions have involved researchers with different personal histories, methodological toolboxes, and countries of origin and employment. We hope that our contribution will inspire more research on how researchers experience and view current funding policies. Ultimately, we need systematic and informed discussion regarding which values to emphasize and deemphasize. We can then use this information to guide decisions regarding allocation constraints and broader policies, with the ultimate goal of generating funding policies that makes sense to all parties involved.

Following this synthesis, we conclude with some recommendations for best funding practices that should foster scientific quality; we have linked these recommendations to the different elements of funding that we have scrutinized in our paper (Table 6). We realize that our comparative study of cross‐national funding schemes and their effect on science is limited by its largely subjective nature. We have reviewed what we, as a collective of evolutionary biologists, encounter when we apply for funding, and we have focused on eleven funding agencies with which we have experience. We thus acknowledge that our review might not be easily extended to all fields or funding agencies. The inferences that we can make are also limited by the fact that we confined our review to funding agencies from Australia, North America and Europe; it would be very useful and interesting for a future analysis to extend to a more diverse sample. Another relevant issue that we have not reviewed, but that deserves consideration, is the degree to which institutional (e.g., internal university) funding is available within and across countries.

**Table 6:** Recommendations for best practices with respect to maximizing scientific quality

Element of funding	Recommended best practices
Societal relevance	Overt emphasis should be on research approach and design rather than project outcome
Top‐down vs. bottom‐up	Fund bottom‐up research that also (when applicable) integrates science in society aspects Reduce top‐down constraints
Applied vs. basic	Set apart substantial explicit funding for basic science
Allocation of money	Mainly small amounts to many researchers Some larger funds to interdisciplinary groups (e.g., excellence centres)
Consortia vs. individual‐led	Fund both consortia and individual‐led projects, which each confer specific and unique benefits
Long‐term vs. short‐term	Fund more long‐term research Provide checkpoints and follow‐ups
Flexibility of funding schemes	Increase flexibility in time, budget, team size with respect to best fit to the research vs. meeting inflexible standards
Acceptance rates and who applies	Provide smart demand management schemes (e.g., limit no. of applications/researcher; size and type budget/researcher; etc.) These schemes should be field/domain‐specific
Who/what is being judged?	Establish different categories: e.g., quality of project for younger researchers; quality of researcher for established researchers
Administrative burden	Reduce and simplify Tailor length of grant sections to evaluation type needed
Who is reviewing	Panel and external experts Invest in the quality of the experts and panel; reviewers from other countries in most smaller countries
Interviews/rebuttals	More rebuttals in general
Mobility	Encourage mobility when reasonable Emphasize flexibility, leaving the possibility to tailor to the individual needs of project/researcher
Diversity	Increase funds, and their diversity, for vulnerable groups Within projects, demands for diversity should fit the project rather than attempt to meet preset standards Within projects, take into account different hierarchy levels within an organization

Finally, we believe that it might be useful to consider whether science would actually benefit from potential optimization, and resulting standardization, of funding schemes across nations. One potential downside of this strategy is that at least some of the existing diversity in funding schemes might be positive in providing a diversity of “niches” in a global “ecosystem” funding setting, allowing researchers to find their optimal niche (i.e., funding scheme). From this perspective, descriptions such as ours of cross‐nation variation in funding schemes might prove useful to individual researchers who have the mobility to move across countries. First and foremost, however, we hope that funders will make use of our comparative overview to critically evaluate and improve their funding schemes.

## AUTHOR CONTRIBUTIONS

The authors contributed to the paper as follows: SM, MN and RB conceived the original idea and JMR and HK subsequently contributed substantially to conceptual development. SM and MN orchestrated discussions and led article development and revision. SM drafted the article, and all authors contributed to constructive discussions, data compilation and article editing.
